# AFruitDB: A comprehensive dataset of six commonly used Asian fruits for advanced grading and biodiversity insights

**DOI:** 10.1016/j.dib.2025.111380

**Published:** 2025-02-11

**Authors:** Mayen Uddin Mojumdar, Shahrin Islam, Md Al Mamun, Rifat Hasan, Shah Md Tanvir Siddiquee, Narayan Ranjan Chakraborty

**Affiliations:** Department of Computer Science and Engineering, Daffodil International University, Daffodil Smart City, Birulia, Dhaka 1216, Bangladesh

**Keywords:** Asian subcontinent, Biodiversity conservation, Deep learning, Fruit classification, Genetic variation, Quality grading, Sustainable agriculture

## Abstract

The Asian subcontinent produces a vast range of fruits throughout the seasons. However, correctly classifying these fruits according to their qualities can be difficult, frequently necessitating the knowledge of fruit experts and cutting-edge equipment to produce accurate results. Therefore, to enable sophisticated grading methods that efficiently sort and evaluate fruit quality based on various characteristics (such as form, color, size, texture, and other crucial parameters), A unique dataset is deployed to support advanced grading systems. This dataset helps researchers explore genetic variation, ecological adaptation, and environmental factors that affect fruit qualities for conservation and sustainable agriculture. Using a mobile camera, these data are personally collected at various times of the day at local markets in Bangladesh that receive optimal sunlight. To create a unique dataset, 6 types of fruit consisting of 3,167 images have been collected. These six different types of fruit: apple, banana, burmese grape, mango, papaya, and tomato were used for quality grading, categorizing them as (i) good, (ii) medium, and (iii) bad. This dataset will help researchers in biodiversity conservation by building efficient machine-learning models and applying machine-learning techniques. Smart fruit grading, classification, and yield prediction automation systems can be built with this dataset.

Specifications Table

**Table:** 

Subject	Computer Science
Specific subject area	Fruits grading, Machine learning, Computer vision, Image processing
Type of data	.jpg
Data collection	3167 images with dimensions of 224 × 224 pixels from various local marketplaces throughout Bangladesh have been gathered. The tomato, papaya, mango, Burmese grape, apple, and banana are the six fruits that we have selected for quality grading. Each of these fruits is divided into three quality grades: good, medium, and bad. These allow us to generate a total of 18 subcategories. These data are collected during the months of July, August, and September 2023 by taking images in a variety of weather situations, including sunny, cloudy, and rainy conditions, as well as at different times of the day, specifically in the morning, noon, and afternoon. Different phone cameras, including the Mi 9T, the iPhone SE, and the Vivo, are used to ensure that all of the images have the same resolution. This dataset is collected from several local marketplaces in Bangladesh, such as Karwan Bazar, Park Bajar, Santosh Bazar, and Jatrabari Bazar. There needed to pre-process the data for fruit grading to enhance visual quality. As some images were captured by iPhone SE, all the pictures are converted to jpg format in the dataset. Fruits are separated from their backgrounds using backdrop removal. The skin tone, ripeness indicators, and surface defects were highlighted through color improvement using Contrast Limited Adaptive Histogram Equalisation (CLAHE). This is crucial to grading since it exposes the subtle differences between ``good,'' ``medium,'' and ``bad.'' After that, all the images are scaled to 224 by 224 pixels, which is consistent and lets us confidently compare and examine fruits of different classes. TensorFlow's ImageDataGenerator handles these pre-processing tasks and generates a refined dataset for accurate fruit grading.
Data source location	Data were collected using mobile phone cameras from the following local markets in Bangladesh: 1. Karwan Bazar, Tejgaon, Dhaka-1225 (Latitude: 23.75218546019505, Longitude: 90.39409484060864), 2. Park Bazar, Tangail Sadar, Tangail-1900 (Latitude: 24.251049783304733, Longitude: 89.91194110302392), 3. Santosh Bazar, University Road, Tangail-1902 (Latitude: 24.2337964036924, Longitude: 89.89024064381567), and 4. Jatrabari Bazar, Dhaka-1232 (Latitude:23.709256640583664, Longitude: 90.43392330454013)
Data accessibility	Repository name: Mendeley data Data identification number: https://data.mendeley.com/datasets/bz65dz2pbj/1 Direct URL to data: 10.17632/bz65dz2pbj.1

## 1 Value of the Data

• This dataset includes six different types of fruit image data that can be used for quality grading. These fruit images are collected from various Bangladeshi local markets and categorized as (a) good, (b) medium, and (c) bad.
• This dataset provides real-world examples of categorizing different fruit species by physical features and quality, such as ripeness and freshness. It is useful for creating machine learning models for agricultural, retail, and quality control applications.
• Using this dataset, it is possible to create an automated system for fruit grading using artificial intelligence. As a result, labour costs can be reduced, and there is no need to contact fruit experts at farms or marketplaces. This dataset will be very useful in establishing a sustainable automated agriculture system.
• Researchers in relevant fields can utilize this dataset to automate fruit grading, quality control, and biodiversity studies through machine learning or other artificial methodologies. All of this information is also useful for farmers, food dealers, food scientists, botanists, biodiversity scientists, and others interested in fruit grading systems.
• This dataset records fruit size, colour, and texture across varieties and grades to reveal biodiversity. Researching how climate, soil, and production practices affect these fruits can help conserve and sustain agriculture by identifying resilient or high-quality varieties.
• Expanding this dataset by including more photos of the existing fruit kinds would increase its robustness and aid in capturing a wider variety of variances, such as differences in size, shape, color, and texture caused by environmental conditions or ripeness stages. Furthermore, including photos of other fruits and vegetables would greatly boost the dataset's richness and usefulness. This update would make the dataset more suited for a broader range of applications, including multi-class classification and object detection.

## 2 Background

Fruits are well-known for their nutritious content and delicious flavour. Asian fruits come in a wide variety, including tropical types that are prized for their flavour and healthful properties. The creation of an accurate and quick automatic system for fruit classification is hampered by the lack of a fruit dataset with consistent images, which is the main constraint in horticultural crops. Fruit storage, processing, and export sectors require accurate and timely classification of fruit based on quality and ripeness. The article provides a comprehensive dataset on banana and guava fruits. The data was categorized into three categories: Class A, Class B, and Defect, based on physiological changes [1], images are categorized based on the fruit's maturity level (mature, half-mature, and mature) [2]. Developed an image dataset of Indian fruits, incorporating quality parameters for those that are widely consumed or exported. Therefore, they selected six fruits: apple, banana, guava, lime, orange, and pomegranate to compile a dataset [3]. A neat and clean dataset is the elementary requirement to build accurate and robust machine learning models for the real-time environment. There is a loss of 30–35 % of the harvested fruit since there aren't enough trained workers. The subjective nature of human vision makes accurate fruit identification, categorization, and grading a challenge. Therefore, the fruit industry must implement an automated system [4,5].

While much of the previous research has focused on certain fruits like bananas or guavas, or popular types in India like apple, guava, lime, orange, or pomegranate, this dataset extends the scope to include six Asian fruits: tomato, papaya, apple, banana, burmese grape, mango, and papaya. By highlighting under-researched fruits like the Burmese grape, this unique assortment highlights regional diversity and addresses gaps in the existing knowledge. Because manual fruit sorting and grading are inefficient and time-consuming. Automatic segregation systems use computer vision and deep learning to reduce human labor, cost, and time [[6], [7], [8]]. Moreover, image processing allows machine-controlled fruit sorting for better quality, productivity, and labor efficiency [9,10].

By using this dataset, researchers can classify fruits and do quality grading across three levels (good, medium, and bad). Unlike most datasets that focus on economically attractive fruit look and maturity, this dataset provides insights into biodiversity. It analyses Asian subcontinent fruits to study genetic and phenotypic variation, improving fruit biodiversity understanding. In Table 1, a clear idea of the present work dataset and others can be noticed. And can easily understand how impactful this dataset is.

**Table 1:** Comparison with existing datasets.

SL	Fruit	Our Dataset	A. Kumari, J. Singh [1]	V. Meshram and K. Patil [3]	S. K. Behera, A. K. Rath, and P. K. Sethy [5]
1	Apple	482	X	2403	X
2	Banana	484	1193	2485	X
3	Burmese Grape	630	X	X	X
4	Mango	618	X	X	X
5	Papaya	451	X	X	300
6	Tomato	502	X	X	X

## 3 Data Description

About 3,167nimages with dimensions of 224 × 224 pixels from several local marketplaces throughout Bangladesh have been collected. For quality evaluation, six types of fruits, tomato, papaya, mango, Burmese grape, apple, and banana, were chosen. To create a total of 18 subcategories, each of the six chosen fruits is separated into three quality grades: good, medium, and bad.

These data were collected in July, August, and September 2023 by taking images in a variety of weather situations, including sunny, cloudy, and rainy conditions, as well as at different times of the day, specifically in the morning, noon, and afternoon. Different types of phone cameras, including the Mi 9T, the iPhone SE, and the Vivo, are used to ensure that all the images have the same resolution. The images of fruits are gathered from several local marketplaces in Bangladesh, including Karwan Bazar (Tejgaon, Dhaka-1225), Park Bajar (Tangail Sadar, Tangail-1900), Santosh Bazar (University Road, Tangail-1902), and Jatrabari Bazar (Dhaka-1232).

Table 2 provides details about the photo collection of this dataset, such as the name of the fruit, the weather conditions at the time of photo capture, the date, the name of the photography device, and the specific location of the photo capture. This detailed information could be used to enhance the understanding of the dataset and its significance to the study.

**Table 2:** Data collection details.

Task	Fruit/Vegetable	Weather	Date	Time	Camera Device	Location
Grading	Apple	Cloudy	3-Aug-23	Afternoon	iPhone SE	Kawran Bazar (Latitude: 23.75218546019505, Longitude: 90.39409484060864)
Grading	Banana	Sunny	8-Jul-23	Morning	Mi 9T	Park Bazar, Tangail (Latitude: 24.251049783304733, Longitude: 89.91194110302392)
Grading	Burmese Grape	Rainy	22-Aug-23	Noon	Vivo	Santosh Bazar (Latitude: 24.2337964036924, Longitude: 89.89024064381567),
Grading	Mango	Sunny	13-Sep-23	Afternoon	iPhone SE	Kawran Bazar (Latitude: 23.75218546019505, Longitude: 90.39409484060864)
Grading	Papaya	Cloudy	27-Jul-23	Morning	Mi 9T	Park Bazar, Tangail (Latitude: 24.251049783304733, Longitude: 89.91194110302392)
Grading	Tomato	Rainy	10-Aug-23	Noon	Vivo	Santosh Bazar (Latitude: 24.2337964036924, Longitude: 89.89024064381567),

Table 3 shows the distribution of images for six commonly used Asian fruits: Apple, Banana, Burmese Grape, Mango, Papaya, and Tomato. Each fruit is categorized into three quality grades: fully fresh (First Grade), another is somewhat fresh (Second Grade), and the last is seriously rotten (Third Grade).

**Table 3:** Dataset Distribution of Quality Grades for Six Asian Fruits.

Class Name		Description	Visualization
Apple	1st grade	This category includes apples that are completely fresh and do not exhibit any obvious signs of damage or rot. These apples have reached their peak condition and are ready to be consumed or sold. The folder labeled in dataset Apple/1st grade has a total of 198 images.	
	2nd grade	This group includes apples with minor imperfections or slight signs of rotting. Although they possess a restricted shelf life, these apples remain fit for consumption or processing. The folder labeled Apple/2nd grade has a total of 172 images.	
	3rd grade	The apples in this category are highly rotten and should not be ingested since they are unsuitable for human consumption. These apples are mostly discarded as waste. The folder labeled in dataset Apple/3rd grade has a total of 112 images.	
Banana	1st grade	In this category, the dataset folder name is Banna/1st grade. It contains 243 images, all completely fresh and in good shape, with no rot or damage symptoms. These images depict bananas suitable for immediate eating or sale.	
	2nd grade	In this category, Bananas have minor imperfections or slight signs of rot. But they are still prepared for consumption or further processing. It basically says the medium condition of banana. This folder's name is Banana/ 2nd grade, and it contains 239 images of bananas.	
	3rd grade	The folder is titled ``Banana/3rd grade'' for this category. This grade includes bananas that have become highly rotten and are inappropriate for consumption. These bananas are normally thrown away as waste.	
Burmese Grape	1st grade	In this category Burmese Grape is considered 1st grade because the folder named Burmese Grape/1st grade includes 308 images of fresh and fully ripe Burmese grapes. So, it can be say the burmese grapes are in good condition.	
	2nd grade	Burmese grapes are here in medium condition, with slight imperfections or early signs of rotting. The dataset's folder name is Burmege Grape/ 2nd grade, including 157 images.	
	3rd grade	This category contains 165 images, all of which are in bad condition. The lowest quality is because the images are heavily rotten and unfit for eating or any type of further processing. The folder name is Burmese Grape / 3rd grade.	
Mango	1st grade	Mangoes that are fully fresh and have no damage or rotten signs are taking place in this category in the folder Mango/1st grade, which includes 251 images of mangoes. That is why they are classified in good condition.	
	2nd grade	This category, which includes,250 images of mangos, is organized into a folder named Mango/2nd grade. This is called second grade because these mango fruits are not fully fresh; some parts have rotten signs. However, they are still of medium quality for processing.	
	3rd grade	Mangoes that are severely spoiled and not suited for human eating make up this grade. Mangoes like these are usually thrown away. The Mango/3rd grade folder contains 117 images of the dataset.	
Papaya	1st grade	These papayas are in peak condition, showing no indications of ripeness or damage. These are of good quality, suitable for both eating and selling. There are a grand total of 152 images in the Papaya/1st grade folder that showcase this particular category.	
	2nd grade	In this category Papaya are not completely fresh, have minor rotting. The folder Papaya/2nd grade contains 147 images representing this category.	
	3rd grade	The folder Papaya/3rd grade, with 152 images representing this 3rd grade. Papayas here are mostly spoiled with rotten signs.	
Tomato	1st grade	The dataset indicates that this 1st grade is in good condition, with tomatoes that are fully fresh without any rotten signs or damage and ready for further processing. The folder name is Tomato/1st grade, and it contains 145 images of tomatoes, all in good condition.	
	2nd grade	Tomatoes that have small flaws or early signs of going bad. These aren't too fresh, but they might still be good for cooking or preparing. The images in this category can be found in the folder Tomato/2nd grade contains 231 images.	
	3rd grade	Totally rotten tomatoes that are not food for humans. These are items that are wasted or should be thrown away. There are 126 pictures for this grade in the folder Tomato/3rd grade.	

## 4 Experimental Design, Materials and Methods

The fruit grading data collection process required several types of materials, as mentioned in Table 2. Table 2 also adds data collection places, weather conditions, and the device used in the working process.

Fruits grading, dataset has 6 types of fruits with three grades of fruits. For grading these three classes, our team set a meeting with a food engineer and collected some important information for the grading process, like how we can grade fruits in these 3 grades and which characteristics are more important for these processes. By considering this information, collected some data for every fruit and examined it by him. Then, finally collected fruit grading datasets from different local markets. Table 4 represents the fruit grading dataset. By seeing this table, find a clear idea of the dataset. In this table, the 1st grade represents fully fresh fruits, the 2nd grade represents some rotten fruits, and finally, the 3rd grade is almost rotten.

**Table 4:** Sample of dataset.

**Fig. 1:**
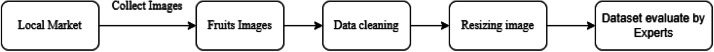
Data collection process of fruits grading dataset.

The fruit grading data collection process followed several steps, as shown in Fig. 1. In this process, some local markets, well-known for fruit selling, were first selected. Then, went to these markets with selected devices. Then, some fruits of each type and grade were collected. Images were captured from the collected fruits. The images were examined to identify which ones needed cleaning or resizing. The images that needed adjustments were cleaned and resized. A food expert checked the processed images. Finally, the verified images were stored.

## Limitations

While collecting samples, we faced some difficulties, especially with the Burmese grape, which was less common due to seasonal availability. This dataset focused on only six types of fruit, which may limit its application in broader markets that handle a wider variety of produce. Local market conditions and mobile cameras may cause unpredictability that affects model consistency under illumination or background factors not in the dataset. Moreover, certain classes have a lower number of fruits; by applying augmentation techniques, researchers can increase the number of samples as required for their machine learning model training.

## Ethics Statement

This dataset was collected ethically from local markets in Bangladesh without harming ecosystems or agricultural practices. It is intended for educational and research purposes, supporting advancements in machine learning and sustainable agriculture. Users must acknowledge the source and avoid misuse for unethical or harmful activities. The goal is to promote responsible innovation and biodiversity conservation.

## CRediT Author Statement

**Mayen Uddin Mojumdar:** Conceptualization, Data Curation, Supervision; **Shahrin Islam:** Software,Writing - Original Draft; **Md Al Mamun:** Project administration, Methodology; **Rifat Hasan:** Data Curation; **Shah Md Tanvir Siddiquee:** Visualization; **Narayan Ranjan Chakraborty:** Writing - Review.

## Data Availability

Mendeley DataA Dataset of Common Asian Fruits for Quality Grading and Biodiversity Research (Original data).
